# *Citrus sudachi* Peel Extract Suppresses Cell Proliferation and Promotes the Differentiation of Keratinocytes through Inhibition of the EGFR–ERK Signaling Pathway

**DOI:** 10.3390/biom10101468

**Published:** 2020-10-21

**Authors:** Shogo Abe, Misako Ueno, Mami Nishitani, Tetsuya Akamatsu, Takumi Sato, Marie Shimoda, Hiroki Kanaoka, Yoshitaka Nii, Hiroko Yamasaki, Keizo Yuasa

**Affiliations:** 1 Department of Biological Science and Technology, Tokushima University Graduate School, 2-1 Minamijosanjima-cho, Tokushima 770-8506, Japan; c501944002@tokushima-u.ac.jp (S.A.); c501934025@tokushima-u.ac.jp (M.N.); 2 Department of Bioscience and Bioindustry, Tokushima University Graduate School, 2-1 Minamijosanjima-cho, Tokushima 770-8513, Japan; c702031004@tokushima-u.ac.jp (M.U.); akamatsu_t@tokushima-u.ac.jp (T.A.); c702032007@tokushima-u.ac.jp (T.S.); 3 Research and Development Department, Matsuyama Co., Ltd., 11-2 Nishibari, Saika-cho, Tokushima 770-8021, Japan; shimoda@matsuyama.co.jp; 4 Research and Development Department, Matsuyama Co., Ltd., 2-17-8 Higashi-sumida, Sumida-ku, Tokyo 131-0042, Japan; kanaoka@matsuyama.co.jp (H.K.); h.yamasaki@matsuyama.co.jp (H.Y.); 5 Food and Biotechnology Division, Tokushima Prefectural Industrial Technology Center, 11-2 Nishibari, Saika-cho, Tokushima 770-8021, Japan; nii@itc.pref.tokushima.jp

**Keywords:** citrus peel extract, keratinocyte, EGF–EGFR–ERK, cell proliferation and differentiation

## Abstract

*Citrus sudachi* is a well-known fruit in Tokushima Prefecture, Japan, and its peels are rich in phytochemicals, including phenolic compounds. Although it is expected that the extract of the *C. sudachi* peel elicits various beneficial physiological activities, the effect on the skin has not been investigated. In this study, we report that the aqueous extract from the peel of *C. sudachi* suppresses cell proliferation of the immortalized human keratinocyte cell line, HaCaT, and primary normal human epidermal keratinocytes. The extract of *C. sudachi* peel suppressed epidermal growth factor (EGF)-induced EGF receptor activation and tumor necrosis factor (TNF)-α-induced extracellular regulated kinase (ERK) 1/2 activation, which suggests that the extract exerts its inhibitory effect through inhibition of both the EGF receptor (EGFR) and its downstream molecules. Additionally, the extract of *C. sudachi* peel potentiated calcium-induced keratinocyte differentiation. These results suggest that the extract of *C. sudachi* peel may have beneficial effects against skin diseases that are characterized by hyperproliferation of epidermal keratinocytes, such as those seen in psoriasis and in cutaneous squamous cell carcinoma.

## 1. Introduction

The skin is a barrier that separates the body from the external environment and plays essential roles in regulating water loss and protecting the body from ultraviolet radiation and microbial infection [1]. The skin is composed of epidermis, dermis, and subcutaneous tissue layers. The epidermis forms the outermost layer of the skin. Keratinocytes are the major cellular component of the epidermis (~90%) and differentiate sequentially from the stratum basale to the stratum spinosum, stratum granulosum, stratum lucidum, and stratum corneum. During differentiation, keratinocytes express specific genes in distinct layers of the epidermis. For example, the expression of keratin 1 and keratin 10 is promoted in the stratum spinosum (early differentiation markers), whereas that of filaggrin is primarily detected in the stratum granulosum [2]. A continuous steady state of proliferation and differentiation of keratinocytes results in the constant renewal of the epidermis, providing an effective barrier for the skin. Disruption of this balance between proliferation and differentiation in keratinocytes leads to skin disorders, such as psoriasis, atopic dermatitis, and ichthyosis vulgaris [2,3].

Keratinocyte proliferation and differentiation are well controlled by the mitogen-activated protein kinase (MAPK) family, including extracellular regulated kinases 1/2 (ERK1/2) and p38 mitogen-activated protein kinase (p38 MAPK). The ERK pathway promotes cell proliferation and regulates cell survival, while the p38 MAPK pathway induces apoptosis and differentiation [4]. ERK signaling is involved in a wide variety of cellular responses, including not only cell proliferation and survival (anti-apoptotic activity) but also differentiation and migration [5,6]. A previous study showed that the transcription factor Elk-1 activated by ERK1/2 induces expression of Egr-1 which promotes cell proliferation and suppresses apoptosis in keratinocytes [7]. Moreover, it has been reported that the expression of involucrin, a keratinocyte differentiation marker, in response to calcium is sensitive to inhibition of the ERK pathway [8], and that ERK1/2 regulates cell migration of keratinocytes through the activation of focal adhesion kinase [9]. Additionally, oncogenic Ras has been shown to be involved in the growth and development of human cutaneous squamous cell carcinoma (cSCC) through the activation of the ERK pathway [10]. Therefore, the search for plant extracts and natural products that could modulate the ERK pathway may lead to the development of new drugs for skin disorders caused by the disruption of the balance between keratinocyte proliferation and differentiation.

*Citrus sudachi* is mainly produced in Tokushima Prefecture, Japan, and is included in the flavorful acid citrus fruit group. Around half of the total production of *C. sudachi* is consumed as fresh fruit, and the other half is consumed as processed products, such as juices. The citrus peel waste of the juice extraction process contains phytochemicals such as vitamins, minerals, flavonoids, coumarin, limonoids, carotenoids, and pectin, which possess a variety of biological functions including antioxidant, anti-inflammatory, antimutagenic, anticarcinogenic, and antiaging properties [11]. Therefore, it has potential for use in the food, cosmetic, and pharmaceutical industries. Since the peel of *C. sudachi* also contains many phenolic compounds, such as hesperidin, naringin, narirutin, and sudachitin (Table 1), which have anti-inflammatory and antioxidant properties [12,13], it is expected that the extract of *C. sudachi* peel has various beneficial physiological properties. Recently, it was reported that the extracts of *C. sudachi* peel attenuate body weight gain in mice fed a high-fat diet and improve lipid metabolism [14,15]. However, the effect of *C. sudachi* peel extract on skin has not been investigated.

In this study, we determined the effect of *C. sudachi* peel extract (SPE) on keratinocyte proliferation and differentiation. We found that SPE suppressed epidermal growth factor (EGF)-induced cell proliferation through inhibition of the ERK pathway in the immortalized human keratinocyte cell line, HaCaT, and primary normal human epidermal keratinocytes. Furthermore, we revealed that SPE enhanced calcium-induced keratinocyte differentiation of HaCaT cells and primary keratinocytes. These results show that SPE possesses antiproliferation and pro-differentiation activities in epidermal keratinocytes, and that it may have the potential for treating skin diseases, such as psoriasis and cSCC, by improving the abnormalities of proliferation and differentiation in keratinocytes.

## 2. Materials and Methods

### 2.1. Preparation of Aqueous Extract of Citrus Sudachi Peel

First, 15 g of crushed *C. sudachi* peel was immersed into 135 mL of distilled water at room temperature for 1 h. The extract was filtered through a 0.45 μm filter, and 1,3-butylene glycol (BG) was added to make a final concentration of 30%, which was designated as the *C. sudachi* peel extract (SPE). The total polyphenol content of SPE was determined using the Folin–Ciocalteu method with gallic acid as a standard, and the content was 2.6 mM gallic acid equivalent. In experiments using SPE, a 30% BG solution in water was used as a negative control.

### 2.2. Phenolic Compound Evaluation by High-Performance Liquid Chromatography (HPLC)

The aqueous extract of *C. sudachi* peel was mixed with a methanol/dimethyl sulfoxide (DMSO) (1:1, *v*/*v*) solution followed by 3 N HCl. After incubation at 70 °C for 1 h, the mixture was filtered through a 0.45 μm membrane filter and analyzed by reverse-phase high-performance liquid chromatography (HPLC) (Wakosil-5C18RS, 4.6 × 250 mm) (FUJIFILM Wako Pure Chemical Corp., Osaka, Japan). The elution solvents used were (A) acetonitrile/50 mM sodium dihydrogen phosphate (pH 2.3) (12:88, *v*/*v*) and (B) acetonitrile/50 mM sodium dihydrogen phosphate (pH 2.3) (60:40, *v*/*v*). Gradient elution was carried out as follows: 0–5 min 100% A, 5–21 min linear gradient to 40% B, 21–23 min 40% B, 23–45 min linear gradient to 100% B, 45–64 min 100% B, and 65–70 min linear gradient back to 100% A (initial conditions). The column temperature was 40 °C, the flow rate was 1.0 mL/min, and the injection volume was 10 µL. The phenolic compounds were identified by the retention time and ultraviolet (UV) spectra of a standard measured from the peak area at 340 nm (Table 1 and Figure S1, Supplementary Materials).

### 2.3. Cell Culture and Induction of Differentiation

Human adult low-calcium high-temperature (HaCaT) cells, from an immortalized human keratinocyte cell line developed by the German Cancer Research Center (Deutsches Krebsforschungszentrum, DKFZ) [16], were cultured in Dulbecco’s Modified Eagle’s Medium (DMEM) (FUJIFILM Wako Pure Chemical Corp., Osaka, Japan) supplemented with 10% fetal bovine serum (FBS) (MP Biomedicals Inc., Irvine, CA, USA), 100 units/mL penicillin, and 100 µg/mL streptomycin. Primary normal human epidermal keratinocytes (NHEKs) were purchased from PromoCell (Heidelberg, Germany). The cells were cultured in keratinocyte growth medium 2 (PromoCell) supplemented with 100 units/mL penicillin, 100 µg/mL streptomycin, and supplement mix (PromoCell) including 0.004 mL/mL bovine pituitary extract, 0.125 ng/mL epidermal growth factor, and 0.06 mM CaCl_2_. To induce differentiation, HaCaT cells and NHEKs were cultured in calcium-free DMEM (Nacalai Tesque Inc., Kyoto, Japan) and keratinocyte growth medium 2 containing 0.06 mM CaCl_2_, respectively, and they were treated with 2 mM CaCl_2_ for 24 h or 72 h.

Human EGF (Peptide Institute, Inc., Osaka, Japan) and human tumor necrosis factor (TNF)-α (Roche Diagnostics GmbH, Mannheim, Germany) were dissolved in phosphate-buffered saline (PBS). Hesperidin, naringin, narirutin (Cayman Chemical Co., Ann Arbor, MI, USA), and sudachitin (FUJIFILM Wako Pure Chemical Corp., Osaka, Japan) were dissolved in 100% DMSO and added to the cell culture medium at a final concentration of 0.1% DMSO.

### 2.4. Lactate Dehydrogenase (LDH) Assay

The activity of LDH released from the cells to the medium was measured using a Cytotoxicity LDH assay Kit-WST (Dojindo Laboratories Co., Ltd., Kumamoto, Japan) according to the manufacturer’s protocol. In brief, HaCaT cells were plated at a density of 7 × 10^3^ cells per well on a 96-well plate and were treated with 0%, 1%, 3%, and 5% SPE for 24 h. As a positive control for strong cytotoxic activity, lysis buffer was added. Working solution was added to each well, and the cells were cultured for 30 min at room temperature in the dark. After adding the stop solution, the absorbance at 490 nm was measured using the Infinite M200 plate reader (Tecan Japan Co., Ltd., Kanagawa, Japan).

### 2.5. Proliferation Assay

Cell proliferation was measured with the 5-bromo-2′-deoxyuridine (BrdU) incorporation assay using a CycLex Cellular BrdU ELISA Kit Ver.2 (Medical & Biological Laboratories Co., Ltd., Nagoya, Japan) as previously described [17]. In brief, HaCaT cells were plated at a density of 7 × 10^3^ per well on a 96-well plate and were serum-starved for 24 h. Next, the cells were pretreated with 3% SPE for 1 h and were treated with either 3% FBS or 1 nM EGF (diluted with DMEM including 0.5% FBS) for 24 h. For NHEKs, cells were plated at a density of 4 × 10^3^ per well on a 96-well plate. After 48 h, the cells were treated with 3% SPE in keratinocyte growth medium 2 supplemented with supplement mix containing growth factor for 44 h. These treated cells were incubated with BrdU for 2 h. Following incubation with anti-BrdU antibody conjugated with peroxidase and substrate, the absorbance at 450 nm (540 nm as a reference) was measured using the plate reader.

### 2.6. Immunoblot Analysis

Immunoblot analysis was performed as previously described [18]. Total cell lysates were prepared and subjected to immunoblot analysis using the following antibodies: anti-ERK1/2 (1:1000), anti-phospho-ERK1/2 (1:1000), anti-MAPK/ERK kinase 1/2 (MEK1/2) (1:1000), anti-phospho-MEK1/2 (1:1000), anti-phospho-Raf-1 (Ser-338) (1:1000), anti-EGFR (1:1000), anti-phospho-EGFR (Tyr-1068) (1:1000) (Cell Signaling Technology Inc., Beverly, MA, USA), anti-Raf-1 (1:1000) (BD Transduction Laboratories, BD Biosciences Inc., San Jose, CA, USA), or anti-involucrin (1:1000) (Santa Cruz Biotechnology, Inc., Santa Cruz, CA, USA). The luminescent signals were analyzed using an LAS-4000 image analyzer (Fuji Film Co., Tokyo, Japan). Immunoblot band intensities were quantified using ImageJ software (NIH, Bethesda, MD, USA).

### 2.7. Luciferase Reporter Assay

HaCaT cells were transfected with pFR-Luc and pFA2-Elk1 (Agilent Technologies Inc., Santa Clara, CA, USA) together with pCMV-β-gal using FuGENE HD (Promega Corp., Madison, WI, USA) according to the manufacturer’s instructions. After 24 h, the cells were serum-starved for 2 h and treated with 3% SPE for 1 h; then, they were stimulated with 1 nM EGF for an additional 6 h. The cells were lysed and subjected to the luciferase reporter assay. The luciferase and β-galactosidase activities were measured using a Sirius luminometer (Berthold Detection Systems GmbH, Pforzheim, Germany) as previously described [17]. Luciferase activity was normalized to β-galactosidase activity.

### 2.8. Immunofluorescence Analysis

Immunofluorescence analysis was performed as previously described [18] with slight modifications. In brief, HaCaT cells were seeded at a density of 5 × 10^3^ cells per well on a 24-well plate and cultured in calcium-free medium. After pretreatment with 3% SPE for 1 h, cells were treated with 2 mM CaCl_2_ to induce keratinocyte differentiation. After 72 h, cells were fixed in 3.7% formaldehyde, permeabilized with 0.1% Triton X-100, and then blocked in 5% bovine serum albumin. Next, cells were incubated with rabbit anti-E-cadherin antibody (1:200) (Santa Cruz Biotechnology, Inc., Santa Cruz, CA, USA) overnight at 4 °C. After washing with PBS, cells were incubated with Alexa Fluor 488-conjugated goat anti-rabbit immunoglobulin G (IgG) antibody (Molecular Probes, Thermo Fisher Scientific, Waltham, MA, USA) for 1 h. After re-washing with PBS, fluorescent and bright-field images were obtained using an IN Cell Analyzer 6000 system (GE Healthcare UK Ltd., Little Chalfont, Buckinghamshire, UK).

### 2.9. Reverse-Transcription Quantitative Real-Time Polymerase Chain Reaction (RT-qPCR)

Total RNA was isolated from HaCaT cells and NHEKs using ISOGEN II (Nippon Gene Co., Tokyo, Japan) and then reverse-transcribed using ReverTra Ace qPCR RT Master Mix with gDNA Remover (Toyobo Co., Ltd., Osaka, Japan). Synthesized complementary DNA (cDNA) was analyzed by quantitative real-time PCR using StepOnePlus Real-Time PCR Systems (Applied Biosystems Inc., Foster City, CA, USA) with THUNDERBIRD SYBR qPCR Mix (Toyobo Co., Ltd., Osaka, Japan). The sequences of the primers used are shown in Table 2. Glyceraldehyde-3-phosphate dehydrogenase (GAPDH) was used as an internal control.

### 2.10. Statistical Analysis

All experiments were performed multiple times to confirm their reproducibility. One representative set of data is shown in each figure. Data were expressed as the mean ± standard error (SE), and statistical analysis was performed by Student’s *t*-test or one-way analysis of variance (ANOVA) followed by Bonferroni’s multiple comparisons tests using GraphPad Prism software (GraphPad Software, Inc., San Diego, CA, USA).

## 3. Results

### 3.1. Toxicity Evaluation of Citrus sudachi Peel Extract (SPE) on HaCaT Cells

**Figure 1:**
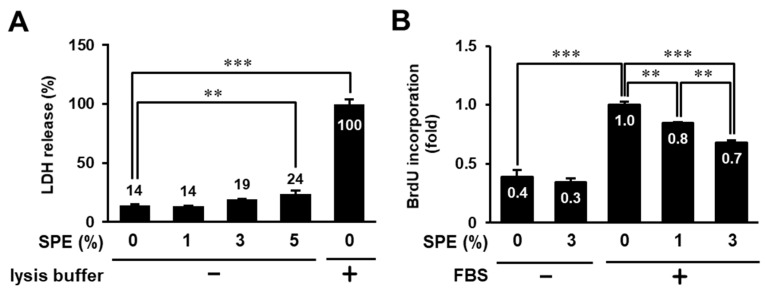
Cytotoxicity and antiproliferation activity of *Citrus sudachi* peel extract (SPE) on keratinocytes. (**A**) HaCaT cells were treated with 0%, 1%, 3%, and 5% SPE for 24 h and subsequently subjected to the lactate dehydrogenase (LDH) release assay. The lysis buffer was added as a positive control. (**B**) Serum-starved HaCaT cells were pretreated with 0%, 1%, and 3% SPE for 1 h, and then incubated in the presence or absence of 3% fetal bovine serum (FBS). After 24 h, the cells were subjected to a 5-bromo-2′-deoxyuridine (BrdU) incorporation assay. The data were expressed as the mean ± standard error (SE) derived from at least three independent experiments, and statistical analysis was performed by ANOVA with Bonferroni’s multiple comparisons test; *** *p* < 0.001, ** *p* < 0.01.

We first examined the cytotoxicity of SPE against human keratinocyte HaCaT cells. The cells were treated with different concentrations of SPE (0%, 1%, 3%, or 5%) for 24 h and then subjected to an LDH leakage assay. As shown in Figure 1A, SPE showed no cytotoxicity at concentrations ≤3% and only very slight cytotoxicity at 5%. Next, we evaluated the effect of SPE on cell proliferation in HaCaT cells using the BrdU incorporation assay. The proliferation of serum-stimulated HaCaT cells was significantly suppressed by SPE (1% and 3%) treatment in a concentration-dependent manner (Figure 1B), suggesting that SPE could suppress cell proliferation of HaCaT cells. Because SPE showed higher antiproliferation activity without cytotoxicity at 3%, we designated 3% as the maximum tolerated concentration for the subsequent experiments.

### 3.2. SPE Suppresses Cell Proliferation and the EGF Receptor–ERK Pathway in HaCaT Cells

**Figure 2:**
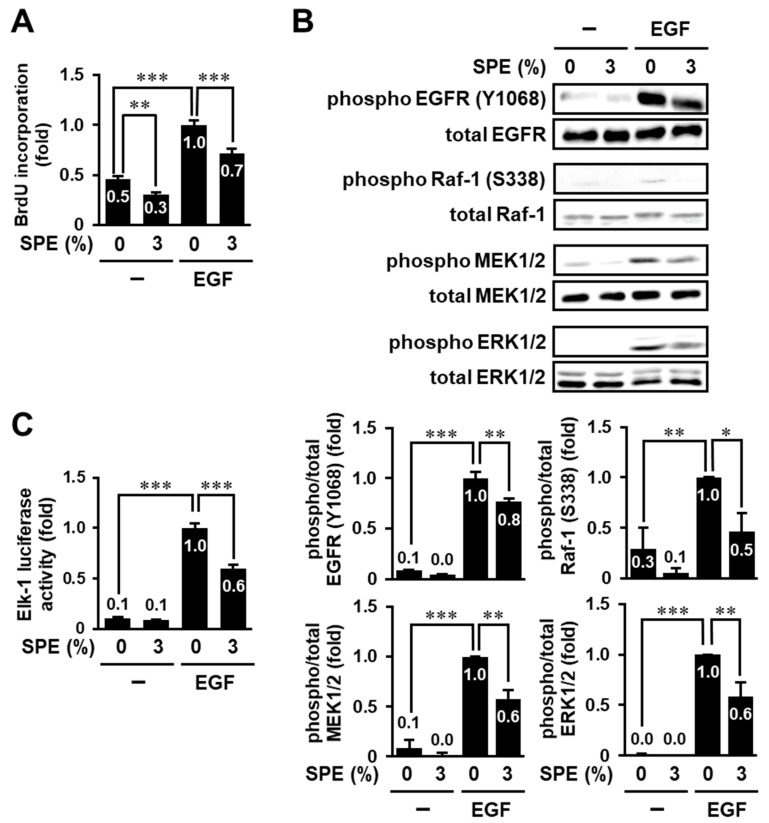
SPE inhibits epidermal growth factor (EGF)-induced cell proliferation and the EGF receptor (EGFR)–extracellular regulated kinase (ERK) pathway in HaCaT cells. (**A**) Serum-starved HaCaT cells were pretreated with 3% SPE for 1 h and subsequently incubated in the presence or absence of 1 nM EGF. After 24 h, the cells were subjected to the BrdU incorporation assay. (**B**) HaCaT cells were serum-starved for 2 h and were treated with 3% SPE for 1 h. Then, the cells were stimulated with 1 nM EGF for 1 h. The cell lysates were analyzed by immunoblot analysis. The levels of phosphorylated proteins were normalized to those of total proteins. (**C**) HaCaT cells were transfected with a GAL4-responsive luciferase reporter plasmid and GAL4–Elk-1 expression plasmid together with pCMV-β-gal. The transfected cells were serum-starved for 2 h and subsequently treated with 3% SPE for 1 h. Then, the cells were stimulated with 1 nM EGF for 6 h and subjected to the luciferase reporter assay. The luciferase activity was normalized to the β-galactosidase activity. The data are expressed as the mean ± SE derived from at least three independent experiments, and statistical analysis was performed by ANOVA with Bonferroni’s multiple comparisons test; *** *p* < 0.001, ** *p* < 0.01, * *p* < 0.05.

We attempted to elucidate the detailed effect of SPE on cell proliferation in HaCaT cells using the BrdU incorporation assay. In keratinocytes, the family of EGF, such as EGF and transforming growth factor-α, promotes cell proliferation, differentiation, and migration, while it inhibits apoptosis through binding of the EGF receptor (EGFR), which is a receptor tyrosine kinase [19]. Hence, we examined whether SPE suppresses EGF-induced cell proliferation of HaCaT cells. The BrdU incorporation assay showed that SPE could significantly attenuate EGF-induced cell proliferation of HaCaT cells (Figure 2A). Furthermore, the in vitro scratch assay indicated that EGF-stimulated cell migration was also significantly inhibited by pretreatment with 3% SPE in HaCaT cells (data not shown).

Cell proliferation is regulated by complex signaling networks, including the MAPK signaling pathway. Activation of the ERK pathway is triggered by the binding of the growth factor to its receptor(s) and is involved in various cellular functions. The ERK signaling pathway involves the sequential activation of Ras, Raf-1, MEK1/2, and ERK1/2. Therefore, we assessed whether SPE suppresses the activation of the ERK pathway, including Raf-1, MEK1/2, and ERK1/2. As shown in Figure 2B, SPE significantly suppressed EGF-induced phosphorylation of Raf-1, MEK1/2, and ERK1/2. A similar result was obtained when using serum as a stimulator instead of EGF (data not shown). In response to the binding of EGF to EGFR, EGFR dimerizes and autophosphorylates at several tyrosine residues, including Tyr-1068, leading to the activation of a network of signaling pathways [19]. Therefore, we examined whether SPE could inhibit EGF-induced EGFR phosphorylation at Tyr-1068. EGF increased EGFR phosphorylation at Tyr-1068, and pretreatment with 3% SPE significantly suppressed EGFR phosphorylation, as well as ERK activation (Figure 2B). Furthermore, we examined the effect of SPE on the activity of Elk-1, a downstream transcription factor of the ERK pathway. The activity of Elk-1 reporter was increased by EGF stimulation, and this increase was significantly inhibited by 3% SPE treatment (Figure 2C). These results suggest that SPE can suppress keratinocyte proliferation through inhibition of the EGF–EGFR–ERK signaling pathway.

### 3.3. SPE Suppresses TNF-α-Induced EGFR-Independent ERK1/2 Activation

**Figure 3:**
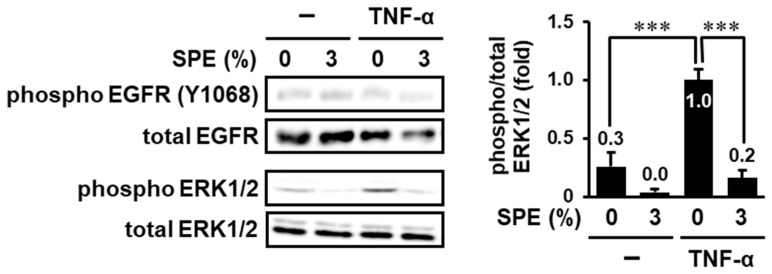
SPE suppresses EGFR-independent activation of ERK1/2 by tumor necrosis factor (TNF)-α. HaCaT cells were serum-starved for 2 h and were treated with 3% SPE for 1 h. Then, the cells were stimulated with 10 ng/mL TNF-α for 1 h. The cell lysates were analyzed by immunoblot analysis. The levels of phosphorylated proteins were normalized to those of total proteins. The data are expressed as the mean ± SE derived from at least three independent experiments, and statistical analysis was performed by ANOVA with Bonferroni’s multiple comparisons test; *** *p* < 0.001.

Although SPE suppressed EGFR phosphorylation on Tyr-1068, the inhibitory efficiency (23%) was less than the efficiency on Raf-1, MEK1/2, and ERK1/2 phosphorylation (41–54%), suggesting that SPE may exert an inhibitory effect through multiple targets (EGF–EGFR and its downstream molecules). Thus, in order to narrow down the target candidates of SPE, we examined the effect of SPE on TNF-α-induced EGFR-independent ERK1/2 activation. As shown in Figure 3, TNF-α did not affect the phosphorylation of EGFR at Tyr-1068. On the other hand, ERK1/2 was significantly activated in response to TNF-α, and 3% SPE almost completely inhibited this increase. These results indicate that SPE can suppress both EGFR and its downstream molecule(s).

### 3.4. SPE Suppresses Cell Proliferation and the ERK Pathway in Normal Human Epidermal Keratinocytes

**Figure 4:**
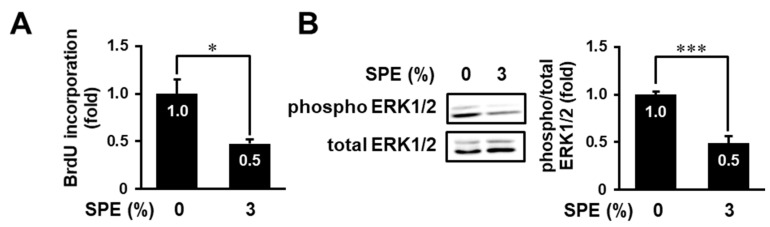
SPE inhibits cell proliferation and ERK1/2 in normal human epidermal keratinocytes (NHEKs). (**A**) NHEKs were treated with 3% SPE for 44 h and were subjected to the BrdU incorporation assay. (**B**) NHEKs were treated with 3% SPE for 1 h. Then, the cell lysates were prepared and immunoblotted with anti-ERK1/2 and anti-phospho-ERK1/2 antibodies. The levels of phosphorylated ERK1/2 were normalized to the levels of the total ERK1/2. The data are expressed as the mean ± SE derived from at least three independent experiments, and statistical analysis was performed by Student’s *t*-test; *** *p* < 0.001, * *p* < 0.05.

Next, we determined the effect of SPE on cell proliferation and ERK1/2 activation in not only an immortalized human keratinocyte cell line, HaCaT, but also primary normal human epidermal keratinocytes (NHEKs). As shown in Figure 4A, the BrdU incorporation assay showed that SPE could significantly attenuate cell proliferation of NHEKs. Similarly, SPE significantly inhibited ERK1/2 phosphorylation in NEHKs (Figure 4B). These data suggest that SPE can also inhibit cell proliferation and ERK1/2 activation in primary normal keratinocytes.

### 3.5. Search for Biologically Active Compound(s) in the Extract of C. sudachi Peel

**Figure 5:**
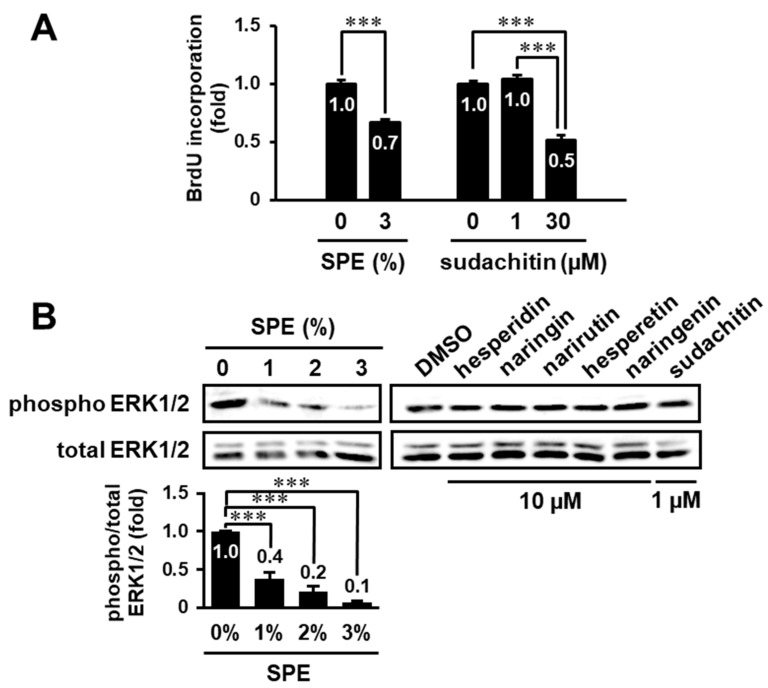
Effects of some phenolic compounds on cell proliferation and ERK1/2 phosphorylation in higher concentrations than those in the extract of *C. sudachi* peels. (**A**) Serum-starved HaCaT cells were pretreated with SPE (3%) or sudachitin (1 or 30 µM) for 1 h and subsequently incubated in the presence of 3% FBS. After 24 h, the cells were subjected to a BrdU incorporation assay. (**B**) HaCaT cells were treated with either 0–3% SPE, dimethyl sulfoxide (DMSO), 10 µM hesperidin, 10 µM naringin, 10 µM narirutin, 10 µM hesperetin, 10 µM naringenin, or 1 µM sudachitin for 1 h. Then, the cell lysates were prepared and immunoblotted with anti-ERK1/2 and anti-phospho-ERK1/2 antibodies. The levels of phosphorylated ERK1/2 were normalized to the levels of the total ERK1/2. All experiments were performed multiple times with similar results. The data are expressed as the mean ± SE derived from at least three independent experiments, and statistical analysis was performed by ANOVA with Bonferroni’s multiple comparisons test; *** *p* < 0.001.

We previously reported that sudachitin, a polymethoxyflavone isolated from the peel of *C. sudachi*, suppressed proliferation and induced apoptosis of HaCaT cells at 30 µM [17]. Thus, we examined whether sudachitin could suppress keratinocyte proliferation at a concentration in 3% SPE (0.46 µM, Table 1). Although 3% SPE significantly inhibited serum-stimulated proliferation of HaCaT cells, sudachitin showed no inhibition of growth even at a higher concentration (1 µM) (Figure 5A). However, in a concentration of 30 µM, it inhibited proliferation, consistent with a previous result [17].

Sudachitin at 30 µM also suppressed the ERK pathway in HaCaT cells [17]. Because the peel of *C. sudachi* contains many phenolic compounds, including hesperidin, naringin, narirutin, and sudachitin (Table 1), we attempted to identify active compound(s) that could affect the ERK pathway in keratinocytes. Although SPE (1–3%) inhibited ERK1/2 phosphorylation in a concentration-dependent manner, hesperidin (10 µM), naringin (10 µM), narirutin (10 µM), or sudachitin (1 µM) individually had no effect on phosphorylation of ERK1/2 even at higher concentrations than those in SPE (Figure 5B). Furthermore, we also examined whether hesperetin (hesperidin aglycone) and naringenin (aglycone of naringin and narirutin) affect ERK1/2 activity, because aglycones generally show a higher permeability than glycosides [20]. However, these aglycones, like their glycosides, had no inhibitory effect (Figure 5B). These results suggest that some known natural products, including hesperidin, naringin, and sudachitin, and/or unidentified compound(s) might additively or synergistically contribute to antiproliferation and inhibitory activity against ERK signaling in keratinocytes.

### 3.6. SPE Potentiates Calcium-Induced Keratinocyte Differentiation of HaCaT Cells

**Figure 6:**
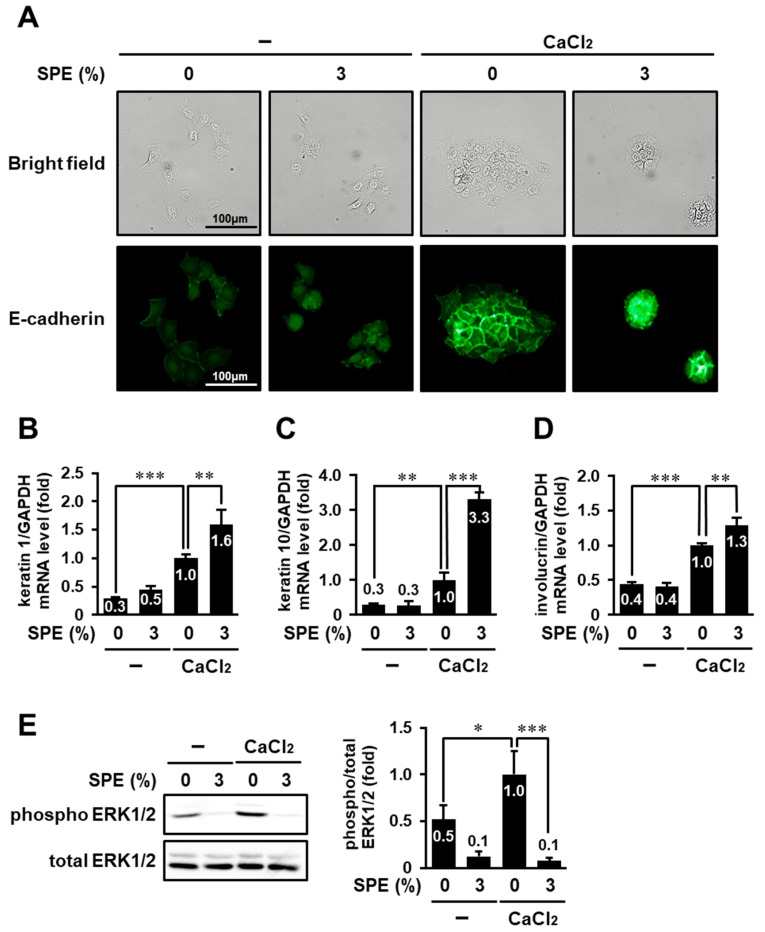
SPE potentiates calcium-induced keratinocyte differentiation in HaCaT cells. (A–D) HaCaT cells cultured in calcium-free medium were treated with 2 mM CaCl_2_ in the presence or absence of 3% SPE for 72 h. The cells were subjected to immunofluorescence analysis with anti-E-cadherin antibody (**A**) and RT-qPCR (**B**–**D**). (**A**) Images were obtained using the IN Cell Analyzer 6000 system. (B–D) The messenger RNA (mRNA) levels of keratin 1 (**B**), keratin 10 (**C**), and involucrin (**D**) were normalized to that of glyceraldehyde-3-phosphate dehydrogenase (GAPDH). (**E**) Serum- and calcium-starved HaCaT cells were pretreated with 3% SPE for 1 h, followed by stimulation with 2 mM CaCl_2_ for 15 min. Then, the cell lysates were analyzed by immunoblotting using anti-ERK1/2 and anti-phospho-ERK1/2 antibodies. The levels of phosphorylated ERK1/2 were normalized to the levels of the total ERK1/2. The data are expressed as the mean ± SE derived from at least three independent experiments, and statistical analysis was performed by ANOVA with Bonferroni’s multiple comparisons test; *** *p* < 0.001, ** *p* < 0.01, * *p* < 0.05.

In addition to hyperproliferation, altered keratinocyte differentiation is also a characteristic of several skin diseases, including skin cancer and psoriasis [21]. Therefore, we examined whether SPE could also affect keratinocyte differentiation. Since differentiation of HaCaT cells is induced by switching from a low-to high-calcium medium [22], HaCaT cells cultured in calcium-free medium were treated with 3% SPE in the presence or absence of 2 mM CaCl_2_. Then, their cell morphology was monitored (Figure 6A). In calcium-free conditions, the cells lost tight intercellular junctions and exhibited a spindle-shaped morphology (upper most left panel). On the other hand, in the presence of calcium, the cells aggregated, formed strong intercellular junctions, and exhibited a cuboid shape (upper second panel from the right), consistent with a previous study [23]. This morphological change was more pronounced when cells were treated with SPE together with calcium (uppermost right panel). Additionally, immunocytochemical staining with an antibody against E-cadherin, which mediates cell–cell contact and enhances the expression of keratinocyte differentiation markers [23,24], was performed. E-cadherin was localized on the entire cell surface in calcium-starved cells (lowermost left panel), whereas it preferentially accumulated at cell–cell contacts in calcium-stimulated cells (lower second panel from the right). The translocation of E-cadherin to cell–cell junction sites was enhanced by treatment with 3% SPE (lower most right panel).

Furthermore, the messenger RNA (mRNA) levels of keratinocyte differentiation markers (keratin 1, keratin 10, and involucrin) were determined. Quantitative RT-PCR analysis showed that high calcium increased the expression of keratin1, keratin10, and involucrin in HaCaT cells (Figure 6B–D). Although SPE did not affect in the absence of calcium, SPE significantly enhanced the increase in expression of differentiation markers elicited by high calcium. These results suggest that SPE may synergistically enhance calcium-induced keratinocyte differentiation in HaCaT cells.

We also examined whether SPE affects the ERK1/2 phosphorylation level in calcium-stimulated HaCaT cells. As shown in Figure 6E, the level of ERK1/2 phosphorylation was significantly increased in response to calcium stimulation, and this increase was completely suppressed by 3% SPE treatment.

### 3.7. SPE Enhances Calcium-Induced Differentiation in Normal Human Epidermal Keratinocytes

**Figure 7:**
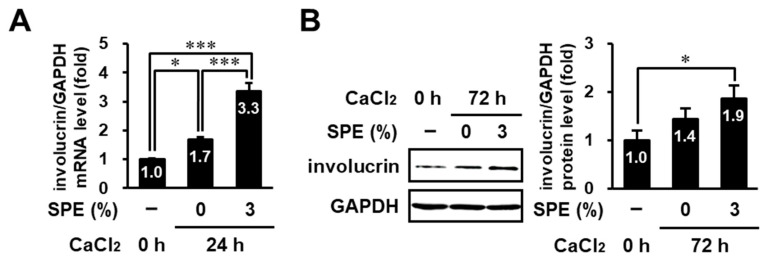
SPE promotes calcium-stimulated differentiation of NHEKs. NHEKs cultured in keratinocyte growth medium 2 including 0.06 mM CaCl_2_ were treated with 2 mM CaCl_2_ in the presence or absence of 3% SPE for 24 h or 72 h. The cells were subjected to RT-qPCR (**A**) and immunoblot analysis (**B**). The mRNA and protein levels of involucrin were normalized to those of GAPDH. The data are expressed as the mean ± SE derived from at least three independent experiments, and statistical analysis was performed by ANOVA with Bonferroni’s multiple comparisons test; *** *p* < 0.001, * *p* < 0.05.

Finally, we examined the effect of SPE on the differentiation in NHEKs. Stimulation with a high concentration of Ca^2+^ (2 mM) resulted in significantly increased involucrin mRNA expression in NHEKs, and 3% SPE induced a further increase in involucrin mRNA expression (Figure 7A). Furthermore, we assessed the protein level of involucrin by immunoblot analysis. The expression of involucrin protein tended to be increased by calcium stimulation and was significantly increased in combination with 3% SPE when compared to the control group (Figure 7B). These results strongly support that SPE can also promote calcium-induced differentiation of keratinocytes.

## 4. Discussion

The ERK pathway (Ras–Raf–MEK–ERK) is involved in the regulation of various cellular processes, such as adhesion, proliferation, cell cycle progression, migration, survival, differentiation, metabolism, and transcription. Previous studies showed that many extracts exert various biological functions by modulating the ERK pathway [25,26]. For example, carrot pentane-based fractions inhibited the proliferation of tumorigenic HaCaT cells through inhibition of the ERK and phosphatidylinositol 3-kinase–Akt pathways, and the extract of *Centella asiatica* induced keratinocyte migration through focal adhesion kinase–Akt, ERK, and p38 MAPK signaling. In this study, we demonstrated that the aqueous extract of *C. sudachi* peel suppresses cell proliferation of HaCaT cells and normal keratinocytes. Furthermore, the extract of *C. sudachi* peel attenuated EGF-induced phosphorylation of EGFR, Raf-1, MEK1/2, and ERK1/2. The development of cancer drugs targeting the EGFR–Ras–Raf–MEK–ERK pathway has been attempted. EGFR tyrosine kinase inhibitors, such as gefitinib, inhibit autophosphorylation by binding to the ATP-binding pocket of the intracellular catalytic kinase domain, resulting in inhibition of the downstream signaling pathways [27]. On the contrary, epigallocatechin-3 gallate, a component of green tea, inhibits the binding of the ligand to EGFR and, subsequently, the dimerization and activation of EGFR [28]. The extract of *C. sudachi* peel may also bind to the ligand-binding site or ATP-binding pocket of EGFR and suppress the autophosphorylation and activation of EGFR. Moreover, the extract of *C. sudachi* peel also suppressed TNF-α-induced ERK pathway activation, although TNF-α did not affect EGFR phosphorylation on Tyr-1068. Previous studies showed that TNF-α stimulates ERK activation through EGFR-dependent or -independent pathways, and that EGFR-independent ERK activation is mediated through the activation of transforming growth factor-β-activated kinase 1 and tumor progression locus 2 [29,30,31]. Taken together, the extract of *C. sudachi* peel may attenuate EGF-induced ERK activation through inhibition of not only EGFR but also its downstream molecule(s), such as Ras and Raf-1, or other signaling molecule(s). Because the combination of drugs targeting different proteins may often increase efficiency and attenuate toxicity [32], the extract of *C. sudachi* peel may be a potential therapeutic avenue for skin diseases caused by abnormalities in the ERK pathway.

We also demonstrated that the extract of *C. sudachi* peel could potentiate calcium-induced keratinocyte differentiation of HaCaT cells and NHEKs. Calcium is involved in the differentiation and proliferation process of keratinocytes [33]. Keratinocytes in low-calcium conditions proliferate but fail to differentiate, and exposure to high calcium levels results in keratinocyte differentiation [22]. Moreover, not only is intracellular calcium concentration a key regulator for keratinocyte differentiation but so is EGFR. EGFR is expressed in all layers of keratinocytes, especially in the basal layer, and a decrease in EGFR expression due to exit the basal layer leads to suppression of proliferation and initiation of differentiation [34]. A previous study showed that both EGFR small interfering RNA (siRNA) and EGFR inhibitors increase the expression of several differentiation markers, including keratin 1, keratin 10, and involucrin in primary human keratinocytes, intact epidermis, and skin squamous cell carcinomas [35]. Furthermore, the expression of differentiation markers was reduced by EGF treatment in a keratinocyte cell line derived from newborn rat skin [36]. Additionally, it was reported that the desmoglein-1/Erbin interaction promoted keratinocyte differentiation by attenuating ERK1/2 activity [37]. On the other hand, a previous study showed that inhibition of MEK1/2, a downstream of EGFR, blocked calcium-induced involucrin expression [8]. In this study, SPE enhanced keratinocyte differentiation only under high-calcium conditions, although SPE inhibited calcium-induced ERK1/2 activation. Therefore, we speculate that SPE may potentiate calcium-stimulated keratinocyte differentiation through the inhibition of EGFR, and further studies are needed to investigate the involvement of the ERK pathway in SPE-enhanced keratinocyte differentiation.

Abnormalities of the EGF–EGFR–ERK signaling pathway in keratinocytes cause skin disorders. For example, EGFR and its ligands are overexpressed in patients with psoriasis, and increased serum EGF concentrations correlate with the severity of psoriasis [38,39]. Overactivation of EGFR caused by a higher concentration of EGF increased epidermal stress and induced remarkable epidermal disorganization with the loss of proper stratification in in vitro human skin equivalents [40]. Furthermore, the ERK pathway is also recognized as an important target for the treatment of cSCC. Oncogenic Ras was shown to be involved in the growth and development of cSCC through the activation of the ERK pathway [10]. EGFR-activating mutations cause constitutive activation of the ERK pathway in cutaneous keratoacanthoma and cSCC [41]. On the other hand, it was reported that a potent Ras inhibitor, salirasib, did not exhibit antitumor activity due to the disability of signaling transmission from Ras to Raf in cSCC [42]. Moreover, mutations in Ras were caused by treating with Raf inhibitor, vemurafenib, in cSCC, and these lesions were blocked by am MEK inhibitor [43]. As the inhibition of the EGFR–ERK pathway is important for the treatment of skin disorders, such as psoriasis and cSCC, further research on mechanisms underlying the inhibition of EGFR–ERK signaling by SPE may lead to the development of therapeutic agents for more effective treatments against skin diseases characterized by abnormalities in the EGFR–ERK signaling pathway.

In this study, we demonstrated that the aqueous extract of *C. sudachi* peel suppresses ERK1/2 phosphorylation. Furthermore, we previously demonstrated that sudachitin, a polymethoxyflavone extracted from the peel of *C. sudachi*, suppresses cell proliferation and migration thorough inhibition of the ERK pathway in HaCaT cells at a relatively high concentration (30 µM) [17]. Therefore, although we examined whether polyphenolic compound(s) included in *C. sudachi* peel could inhibit the ERK pathway, hesperidin, naringin, narirutin, sudachitin, hesperetin (an aglycone of hesperidin), and naringenin (an aglycone of naringin and narirutin) had no effects even at higher concentrations than those in SPE. Additionally, these phenolic compounds, except for sudachitin, failed to suppress ERK1/2 phosphorylation even at high concentration (100 µM) (Figure S2, Supplementary Materials). However, previous studies reported that many flavones such as hesperidin and naringin isolated from citrus peel suppress the ERK1/2 pathway [44,45,46]. For example, hesperidin (approximately 10 µM) found in *C. tangerine* peel suppressed lipopolysaccharide-induced ERK1/2 phosphorylation in HaCaT cells [44], and naringin (100 µM) isolated from *C. grandis* attenuated EGF-induced ERK1/2 phosphorylation in human lung adenocarcinoma cells [45]. Hesperetin also inhibited vascular endothelial growth factor-induced ERK1/2 phosphorylation at 25 µM in human umbilical vascular endothelial cells [46]. On the other hand, it was reported that hesperidin (40 µM), naringin (100 µM), and hesperetin (50 µM) facilitated ERK1/2 phosphorylation in normal human hepatic cells, human bladder carcinoma cells, and murine B16-F10 melanoma cells, respectively [47,48,49]. Therefore, phenolic compounds may regulate the ERK pathway in a cell type-dependent manner, and the phenolic compounds evaluated in this study may have no effect on ERK activity in keratinocytes. Other phenolic compounds contained in *Citrus* peels have been shown to possess ERK1/2 inhibitory activity [50,51]. Tangeretin and quercetin, which are flavonoids obtained from *Citrus* peels, inhibited estradiol-induced ERK1/2 phosphorylation in human mammary ductal carcinoma and suppressed ERK1/2 phosphorylation induced by stimulation of 12-*O*-tetradecanoylphorbol-13-acetate in human breast carcinoma cells, respectively. Taken together with these studies, we speculate that some known natural products, including hesperidin, naringin, and sudachitin, and/or unidentified compound(s) may additively or synergistically affect keratinocyte proliferation and differentiation through regulation of the ERK1/2 pathway. Further studies are needed to identify bioactive compound(s) and investigate the underlying mechanisms of the action.

## 5. Conclusions

The aqueous extract of *C. sudachi* peel suppresses serum- and EGF-induced cell proliferation and enhances differentiation induction in keratinocytes through inhibition of the EGFR–ERK signaling pathway; thus, it may be effectively used for the prevention and treatment of skin diseases, such as psoriasis and cutaneous squamous cell carcinoma, caused by abnormalities in proliferation and differentiation in epidermal keratinocytes.

## Figures and Tables

**Table 1:** Contents of polyphenolic compounds in the extract of *C. sudachi* peel.

Compound	Concentration in Aqueous Extract (µM)	Concentration of SPE Containing 30% BG (µM)	Concentration in 3% SPE (µM)
hesperidin	426	298	8.95
naringin	279	195	5.85
narirutin	286	200	6.01
sudachitin	22	15	0.46

**Table 2:** The list of primers used for RT-qPCR.

Gene	Forward Primer (5’ to 3’)	Reverse Primer (5’ to 3’)
keratin 1	ATATGGGGGTGGTTATGGTCC	GTGACTTGATTTGCTCCCTTTCT
keratin 10	TTGCTGAACAAAACCGCAAAG	GCCAGTTGGGACTGTAGTTCT
involucrin	ACTGAGGGCAGGGGAGAG	TCTGCCTCAGCCTTACTGTG
GAPDH	CAGCCTCAAGATCATCAGCA	CATCCACAGTCTTCTGGGTG

## Supplementary Materials

The following are available online at https://www.mdpi.com/2218-273X/10/10/1468/s1: Figure S1: HPLC chromatogram of the aqueous extract from *C. sudachi* peel; Figure S2: Effects of some phenolic compounds included in SPE on ERK1/2 phosphorylation at the high concentrations.
