# Supplementary material for: Citrus sudachi Peel Extract Suppresses Cell Proliferation and Promotes the Differentiation of Keratinocytes through Inhibition of the EGFR–ERK Signaling Pathway
Source: Biomolecules. 2020 Oct 21;10(10):1468. doi: 10.3390/biom10101468 (PMC7589747; doi:10.3390/biom10101468)
Supplement: Supplementary file 1 [file biomolecules-10-01468-s001.pdf]

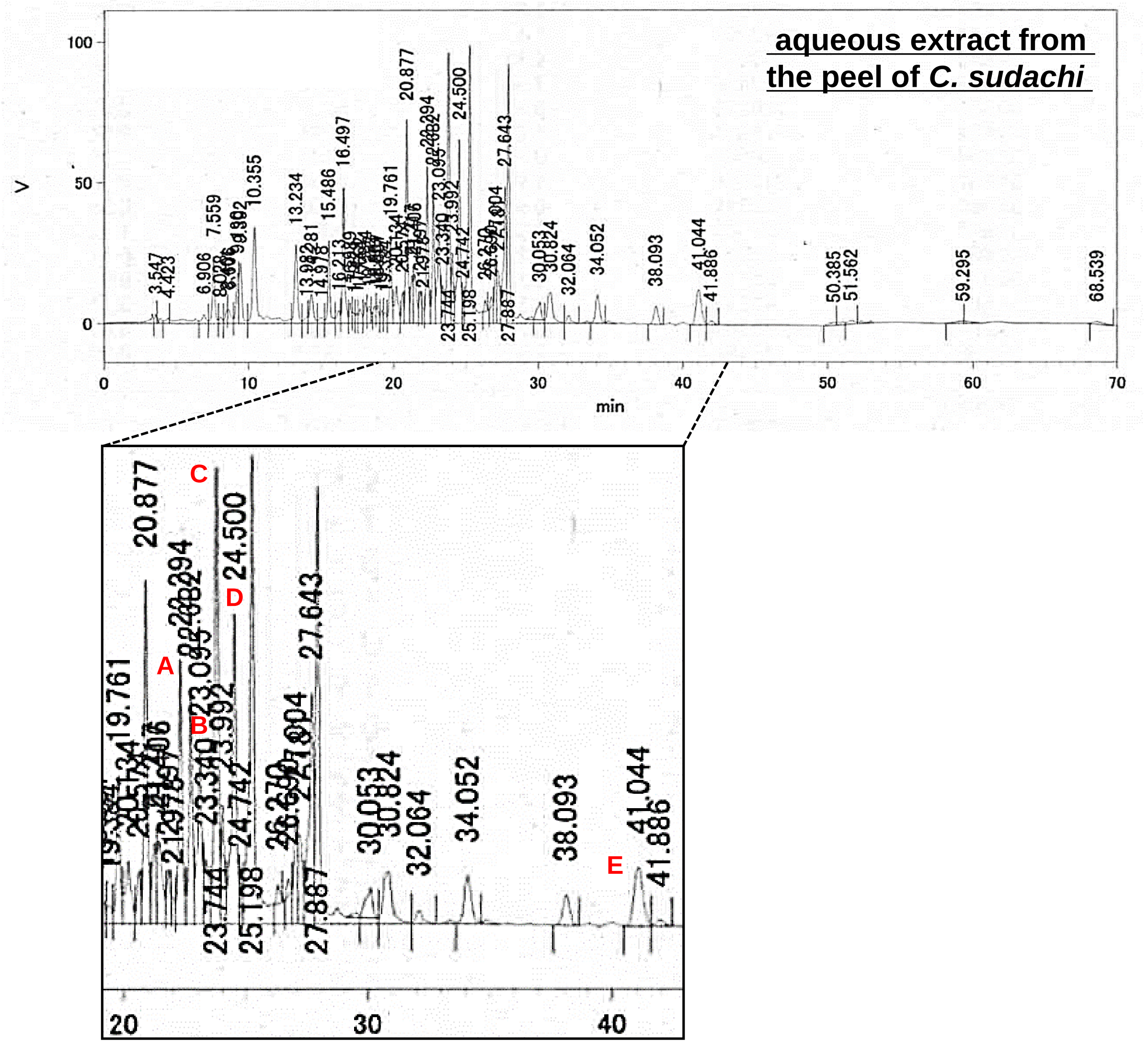

standard compound	retention time (min)	area
narirutin (1.72 mM)	22.245	3385838
naringin (1.72 mM)	23.065	2932094
hesperidin (1.64 mM)	23.782	3665679
neohesperidin (1.64 mM)	24.470	3534963
sudachitin (2.78 mM)	41.113	39898784

HPLC peak	retention time (min)	area
A (narirutin)	22.294	562362
B (naringin)	23.095	474515
C (hesperidin)	23.744	953614
D (neohesperidin)	24.500	824369
E (sudachitin)	41.044	317713

Figure S1. HPLC chromatogram of the aqueous extract from *C. sudachi* peel. The phenolic compounds were identified by direct comparison of their UV spectra and retention times measured from the peak at 340 nm with the standard compounds.

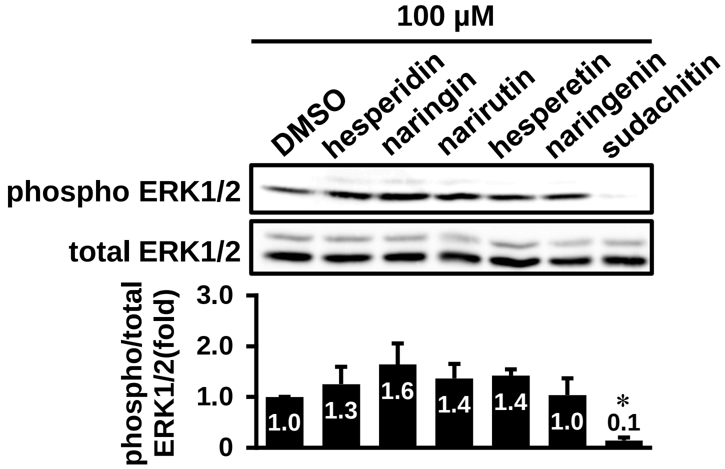

Figure S2. Effects of some phenolic compounds included in SPE on ERK1/2 phosphorylation at the high concentrations. HaCaT cells were treated with 100 µM hesperidin, 100 µM naringin, 100 µM narirutin, 100 µM hesperetin, 100 µM naringenin, or 100 µM sudachitin for 1 h. Then, the cell lysates were prepared and immunoblotted with anti-ERK1/2 and anti-phospho-ERK1/2 antibodies. The levels of phosphorylated ERK1/2 were normalized to the levels of the total ERK1/2. The data were expressed as the mean ± SE derived from at least three independent experiments, and statistical analysis was performed by ANOVA with Bonferroni's multiple comparison test. * $p < 0.05$.
